# Two Cases of Congenital Hypothyroidism Revealing Thyroid Agenesis

**DOI:** 10.3390/medicina59101887

**Published:** 2023-10-23

**Authors:** Leonard Năstase, Octaviana Cristea, Alexandra Diaconu, Silvia-Maria Stoicescu, Ramona Mohora, Bogdan Mihai Pascu, Simona Tania Tala, Ioana Roșca

**Affiliations:** 1 Neonatology Department, National Institute for Mother and Child Health “Alessandrescu-Rusescu”, 011061 Bucharest, Romania; 2 Faculty of Medicine, Carol Davila University of Medicine and Pharmacy, 050474 Bucharest, Romania; 3 Endocrinology Department, National Institute for Mother and Child Health “Alessandrescu-Rusescu”, 020395 Bucharest, Romania; 4 Neonatology Department, Clinical Hospital of Obstetrics and Gynecology “Prof. Dr. P. Sârbu”, 060251 Bucharest, Romania

**Keywords:** congenital hypothyroidism, thyroid agenesis, neonatal hypothyroidism

## Abstract

Congenital hypothyroidism (CH) may have major detrimental effects on growth and neurological development, but early intervention leads to excellent outcomes. CH is classified as transient or permanent, primary or secondary, with primary CH being the most common neonatal endocrine disorder. Most patients with CH do not present any typical signs and symptoms of hypothyroidism shortly after birth, partly due to transplacental maternal thyroid hormone transfer and residual neonatal thyroid function. This paper reports on two CH cases. During the initial Neonatal Intensive Care Unit (NICU) admission phase, CH was not suspected due to nonspecific signs. The distinct characteristics of our cases are as follows: both infants were admitted to the NICU for respiratory distress syndrome, requiring invasive mechanical ventilation, and both were born to diabetic mothers. Following extubation, they both showed similar neurological issues, including reduced muscle tone and feeding difficulties. Initially, those symptoms were attributed to delayed clearance of analgesic and sedative medication. However, symptoms progressively worsened over time. Subsequent tests revealed both meeting CH diagnostic criteria: an unusual ultrasound indicating thyroid agenesis and abnormal hormone levels. Guided by the pediatric endocrinology team, prompt hormonal treatment was started with improvements in neurocognitive function and feeding. Usually, CH screening involves blood samples from healthy newborns at 2–3 days of life. Abnormal results require confirmation, prompting treatment within two weeks. Certain NICU-admitted infants face higher diagnosis delays, as seen in those two cases where CH screening was postponed. Thus, for all neonates with persistent pathologies unresponsive to standard etiological treatment, conducting a comprehensive anamnestic evaluation of the medical history, along with maternal preconceptional and prenatal nutrition, is recommended.

## 1. Introduction

Congenital hypothyroidism (CH) may have major detrimental effects on growth and neurological development, but early intervention can lead to excellent outcomes [1].

CH can be defined as variable dysfunction of the hypothalamic-pituitary-thyroid axis present at birth. This dysfunction leads to insufficient thyroid hormone production and, subsequently, to moderate or severe thyroid dysfunction.

CH is classified into primary—an abnormal thyroid gland development or function, and central—a hypothalamus and pituitary gland pathology leading to thyroid stimulation alterations [2,3]. Primary HC is one of the most common neonatal endocrine pathologies [3]. The underlying mechanisms can be a thyroid development failure (thyroid dysgenesis) or thyroid inability to produce a sufficient amount of hormones, even if it is anatomically normal (dyshormonogenesis) [4].

The prevalence of the disease in the population depends on the screening test strategy; thus, the incidence is estimated between 1:3000 and 1:2000 for primary HC and 1:16,000 for central HC [2]. The latest report from the INSMC regional center in Bucharest, Romania indicates one confirmed case of primary HC among 3576 live newborns [5].

The majority of newborns affected by HC are unlikely to exhibit early symptoms of postnatal impairment. This is believed to be attributed to the transplacental transfer of maternal thyroid hormones and the residual thyroid activity in neonates. Classic clinical signs appear over time and include macroglossia, facial edema, umbilical hernia, hypotonia, hypothermia, lethargy [6], goiter, feeding difficulties, constipation, bradycardia, wide fontanelles, prolonged jaundice, and developmental delay. In particular, prolonged indirect hyperbilirubinemia, enlarged posterior fontanel (>0.5 cm), stunted growth, and goitre should raise the suspicion of HC. Universal neonatal screening is very important in the diagnosis of CH. It is generally performed in the first days of life (24–72 h) [4]—at that age the clinical diagnosis of CH is difficult or even impossible [6]. International guidelines recommend immediate initiation of treatment with levothyroxine (LT4) and closely monitoring hormonal levels to facilitate appropriate adjustments in therapy [2].

## 2. Materials and Methods

We present two case studies involving newborns who were patients at the Neonatology Department, Polizu Maternity and later at the Pediatric Endocrinology Department, both within INSMC “Alessandrescu-Rusescu”. During their hospitalization, both cases were diagnosed with CH. These infants were full-term and came from pregnancies that were investigated. They were delivered via cesarean section, cranial presentation. Although resuscitation was not required at birth, they later needed specialized intensive care and invasive respiratory support. Initially, their progression showed non-specific and atypical symptoms, resembling respiratory distress syndrome. As time passed and without sedation, the neurological manifestations persisted, prompting further investigation.

After laboratory examinations in both cases, CH was detected, leading to the initiation of hormone replacement treatment in collaboration with the endocrinologist. The patients showed positive progress, experiencing a rapid improvement in neurological status. They remained under the close supervision of the pediatric endocrinology team for a period of 6 months.

## 3. Cases Presentation

Case 1—A female newborn, gestational age 37 weeks, with a birth weight of 2950 g, was delivered via cesarean section due to a lack of labor progression. She was appropriate for gestational age (AGA) and had a normal ponderal index. The mother, a primigravida primipara (1G1P), had a history of gestational diabetes controlled with nutrition therapy, ongoing hormone replacement treatment for autoimmune thyroiditis, and the detection of polyhydramnios during pregnancy.

Shortly after birth, the newborn was admitted to the NICU due to functional respiratory syndrome. Despite initial oxygen therapy, her condition did not improve. Non-invasive respiratory support (HHHFNC/IPPVn/HFOVn) was initiated and progressed to invasive mechanical ventilation (endotracheal intubation) due to a pneumothorax. While her respiratory condition improved, she continued to display muscle hypotonia and hyporeactivity, even without analgesia or sedation.

Furthermore, she exhibited a distended abdomen, sensitivity to palpation, visible intestinal loops, frequent emesis, regurgitation, and altered stool appearance. The suspicion of neonatal sepsis arose due to the clinical and laboratory picture, confirmed by a positive blood culture for Candida albicans. Specific therapy was initiated, along with a lactose-free, extensively hydrolyzed protein formula for nutrition.

Despite these interventions, neurological issues persisted, including hypotonia, absence of sucking reflex, and velo-palatine incoordination. These challenges raised suspicions of a metabolic or an endocrine disorder, leading to interdisciplinary consultations. (Table 1).

Consequently, laboratory investigations, imaging, and an endocrinology consultation were conducted. The results revealed high TSH (>75.0 Uui/mL), low T3 (<1.00 pg/mL), and low T4 (0.96 ng/dL), along with the absence of thyroid tissue on ultrasound examination. The correlation of clinical and paraclinical data led to a diagnosis of CH. As a result, specific hormone replacement therapy was initiated on day 42 with a dose of 10 mcg/kg, leading to TSH normalization after 14 days (Table 2).

The patient was clinically and paraclinically monitored at 2-week intervals until the 6th month, and subsequently at monthly intervals. During this period, muscular hypotonia resolved, the skin regained its normal color, hydration and digestive improvement was achieved with an end to vomiting and normalized stools, and weight was corrected, reaching growth curves from the 10th to the 50th percentile. Thyroid ultrasound detected a thyroid lodge where, given the patient’s age and residual infiltrate, thyroid tissue in the form of 2 lobes and an isthmus could not be detected. This was repeated at 6 months, when the diagnosis of CH due to thyroid agenesis was confirmed. Throughout these months, the treatment dose was adjusted according to the child’s weight gain and paraclinical evidence of thyroid function. Starting from an optimal dose of soluble LT4 at 10 mcg/kg with a decreasing trend, the patient is currently receiving a dose of 2 mcg/kg, with a balanced thyroid function for approximately 3 months (Table 3).

Case 2—A male newborn, gestational age 37 weeks, with a birth weight of 3260 g, appropriate for gestational age (AGA), and normal ponderal index. He was delivered by cesarean section due to uterine scarring. Regarding maternal history, the mother, a Gravida 3 Para 3 (3G3P), has insulin-dependent gestational diabetes, history of electric conversion for paroxysmal supraventricular tachycardia before pregnancy, and a smoking history of 5 cigarettes per day since the age of 15. Otherwise, there were no notable changes during pregnancy.

Immediately after birth, the newborn was admitted to the NICU for erythroderma, peri-oro-nasal, and extremity cyanosis, and intermittent moaning while exhaling. He was transferred to the NICU for fluid balance and oxygen therapy. His progress was unfavorable, with worsening respiratory distress syndrome. Non-invasive respiratory support (HFNC, nCPAP, nIPPV) was initiated, but without improvement. Therefore, after 48 h of life, intubation, invasive respiratory support (SIPPV), and surfactant administration were required. Lung dysfunction due to maternal diabetes was suspected.

Progress was slow, allowing the discontinuation of invasive mechanical ventilation after 4 days and weaning from oxygen therapy at 20 days of life. He presented upper abdominal distension and multiple bilious emesis. Clinical evolution and positive inflammatory markers supported a high probability of an infection, leading to antibiotic therapy adjustment. Despite these efforts, digestive tolerance remained unsatisfactory, sucking was inefficient with tongue protrusion, and his weight curve remained stationary. Moreover, he exhibited diminished tone and reactivity without sedation and analgesia.

At this point, suspicion of a metabolic or endocrine disorder arose. Other associated disorders or malformations were ruled out through interdisciplinary consultations (cardiology, ophthalmology, and neurology). CH was confirmed through biological tests—high TSH (>75.00 Uui/mL), low FT4 (<0.30 ng/dL), and endocrinology consultation.

Specific treatment was initiated on day 17, with a dose of 10 mcg/kg, leading to TSH normalization after 17 days (Table 2). The infant was clinically and paraclinically monitored at 2-week intervals until the 6th month, and subsequently at monthly intervals. During this period, muscular tone normalized, macroglossia resolved, digestive tolerance improved, vomiting ceased, and appetite increased, leading to rapid weight correction, reaching growth curves from the 10th to the 75th percentile. Immediately after initiating soluble LT4 treatment, the infant gained approximately 800 g in a week, necessitating constant dose adjustments and highlighting the importance of close monitoring for these patients. Starting from an optimal dose of 10 mcg/kg with a decreasing trend, the patient is currently on a dose of 3.2 mcg/kg with balanced thyroid function for approximately 2 months (Table 3).

Thyroid ultrasound detected a thyroid lodge where, given the infant’s age and psychomotor agitation, thyroid tissue could not be identified. This was repeated at 6 months, when the diagnosis of CH due to thyroid agenesis was confirmed.

**Figure 1:**
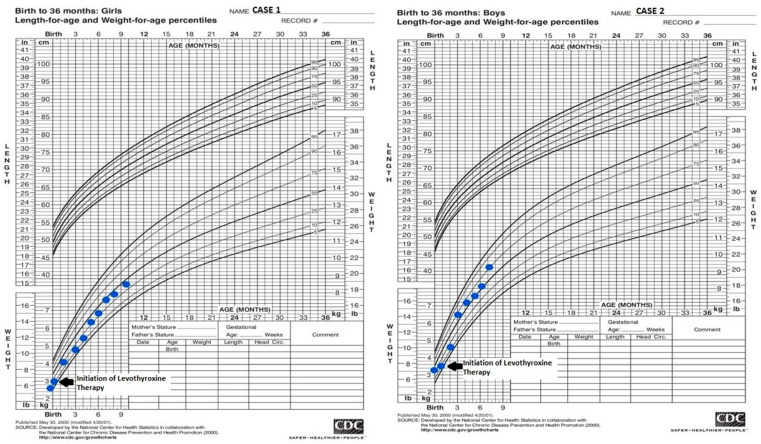
Weight-for-age-percentiles for both cases.

In both cases, after undergoing treatment and therapeutic adjustments based on routine hormonal monitoring, a positive evolution was observed. This included the normalization of muscle tone, improvement in nutrition, and a progressive increase in weight (Figure 1).

Upon discharge from the maternity unit, the newborns were enrolled in a comprehensive multidisciplinary follow-up program. This program involved regular consultations with neonatologists, pediatricians, neurologists, ophthalmologists, and endocrinologists.

## 4. Discussion

### 4.1. Diagnostic Methods

Neonatal screening is essential for the early diagnosis of CH. A blood sample is collected from the newborn’s heel on a card that is sent to the laboratory for analysis. Most screening programs measure TSH levels. Even though it has high sensitivity, HC screening can have up to 10% false negative results. One of the most important causes of false negative results is the delayed rise in TSH levels that often occurs during the first weeks of life. A typical example involves preterm VLBW (<1500 g) infants and neonates admitted to the neonatal intensive care unit [4]. Additionally, the use of glucocorticoids has been observed to inhibit TSH release, as indicated by studies. Somatostatin and dopamine also contribute to this phenomenon [7]. Monozygotic twins present another unique situation, where the normal thyroid function of one twin compensates for the hypothyroidism of the other one intrauterinely [4]. A distinct group at higher risk for CH comprises patients with Down syndrome. For this population, it is advisable to conduct retesting after the completion of the neonatal period [2]. Abnormal screening results should be confirmed by serum TSH and FT4 testing as soon as possible [4]. The diagnosis of primary HC is confirmed by the presence of a low T4 level and an elevated TSH level (>20 mIU/L) and requires the institution treatment with LT4 (10–15 µg/kg/day) [2,6].

### 4.2. CH Types

The majority of CH cases are caused by gland formation defects (85% of cases) and hormonal synthesis defects (15%). In rare cases, other factors are involved—maternal antibodies that block the TSH receptor, and deficiency or excess of iodine. Recent data also suggest a genetic cause of dysmorphogenesis in some patients [2,8].

#### 4.2.1. Primary CH

Most cases of Primary CH are caused by a thyroid dysgenesis. These are sporadic cases and the cause of occurrence is in most cases unknown. In a small proportion, a mutation in one of the many genes involved in the thyroid gland development may be present [4]. Thyroid dysgenesis has maintained a constant incidence over the past 25 years, but the incidence of dyshormogenesis has risen sharply. Neonatal hypothyroidism with a eutopic thyroid gland can be caused by a multitude of extrinsic factors.

a. Antithyroid medication such as methimazole or propythilouracil is commonly administered during pregnancy to manage maternal hyperthyroidism. These medications have the ability to cross the placenta, potentially leading to the development of transient CH [4]. This occurrence is attributed to the medications’ capacity to hinder the synthesis of fetal thyroid hormones [6]. The effects persist until the substances are metabolized and eliminated from the newborn’s circulation [4]. It’s important to note that the use of antithyroid medications during pregnancy requires careful consideration due to their potential impact on fetal thyroid function and the subsequent risk of transient CH. Monitoring and appropriate medical management are crucial to ensure the well-being of both the mother and the developing fetus.
b. Maternal autoimmune thyroid disease, including Graves’ disease, involves the presence of IgG antibodies that can effectively block the activation of the TSH receptor. These antibodies have the ability to traverse the placental barrier, potentially leading to the development of transient CH. It is worth noting that this condition might require a period of 2 to 6 months for resolution [4,6]. The interplay between maternal autoimmune thyroid disease and its impact on the developing fetus underscores the complexity of maternal-fetal health dynamics. The ability of maternal antibodies to cross the placenta and influence fetal thyroid function emphasizes the importance of vigilant monitoring and timely intervention when required. As with any medical condition during pregnancy, thorough assessment and management are essential to ensure the optimal health of both the mother and the newborn.
c. Iodine deficiency or excess: It is well known that iodine is an essential constituent of thyroid hormones, and its deficiency represents a global cause of HC [4], especially in endemic areas [6]. Universal salt iodization was promoted at the World Summit for Children in New York in 1990, with the aim of eliminating iodine deficiency. In Romania, continuous iodine prophylaxis has been implemented since 2003 due to presence of endemic goiter in various forms in 2/3 of the country’s surface. Iodine deficiency is of particular concern for pregnant women due to their heightened requirement—250 µg/day compared to 150 µg/day outside of pregnancy. A study from our country concluded that, after 10 years from the implementation of continuous iodized salt, pregnant women have a normal iodine status with or without iodine supplements [9]. Premature infants are more susceptible to iodine deficiency compared to full-term infants due to the shortened period of maternal-fetal iodine transfer. During pregnancy, the developing fetus relies on the mother’s thyroid hormones for proper brain and nervous system development. Iodine is an essential component of these hormones [10]. Premature infants often have underdeveloped thyroid hormone processes, including hormonogenesis (production of thyroid hormones) and the hypothalamic-pituitary-thyroid axis (the hormonal feedback system regulating thyroid function). The conversion of the inactive thyroid hormone T4 to the active T3 form is not fully efficient in premature infants, further affecting their thyroid function [6]. In addition, they often require parenteral nutrition due to their underdeveloped digestive and special formulas for their nutritional needs systems, which might not always provide sufficient iodine, exacerbating the risk of deficiency [4]. On the other hand, excess iodine physiologically causes a decrease in thyroid synthesis that is often transient [4]. This phenomenon is known as the Wolff-Chaikoff effect. It occurs when the thyroid gland becomes exposed to a sudden high concentration of iodine, leading to a temporary inhibition of thyroid hormone production. In particular, both the fetus and the newborn are very sensitive to excess iodine [6]. The premature thyroid acquires the ability to recover from the Wolff-Chaikoff effect only at 36–40 gestational weeks; therefore premature infants remain a group at increased risk of hypothyroidism. This excess iodine can be caused by iodine-based antiseptics [4], increased cutaneous absorption and decreased renal excretion of iodine [6], or excessive maternal iodine intake (diet or supplements) that is transmitted to the newborn through breastfeeding [4]. Excessive iodine consumption can be easily estimated by documenting the use of food supplements, but it is more difficult to estimate the dietary iodine intake. A thorough maternal history may reveal certain dietary habits, with an increased consumption of iodine-rich foods (seaweed, fish, and seafood) [11]. A case of HC caused by excessive maternal Iodine intake through constant and sustained consumption of seaweed has been described in the literature, leading to increased excretion of iodine in breast milk and subsequently, to a high port of iodine in the newborn [12].
d. Other causes: Congenital hepatic hemangiomas may produce large amounts of type 3 iodothyronine deiodinase, a major physiologic inactivator of thyroid hormone, leading to the need for higher T4 levels for normal thyroid function [6].

#### 4.2.2. Central CH (Secondary)

Central CH (Secondary) is rare, with recent studies estimating an incidence of 1 in 16,000 newborns. Congenital defects of the thyroid axis typically arise from structural or developmental alterations in the hypothalamus or pituitary gland. These defects often impact not only the thyroid hormone axis but also the pituitary hormone axis, including hormones such as growth hormone, prolactin, adenocorticotropin, and gonadotropins. Some of these conditions have a genetic basis, while others can emerge, often transiently, in newborns exposed to maternal hyperthyroidism during intrauterine development [4].

a. Maternal hyperthyroidism can lead to transient hypothalamic-pituitary suppression in the developing fetus, sometimes necessitating postnatal treatment.
b. Prematurity: Hypothyroxinemia in the presence of a normal TSH level is most commonly observed in premature infants. Additionally, these infants might be exposed to medications that suppress the hypothalamic-pituitary-thyroid axis, such as steroids, dopamine, aminophylline, and caffeine [6].

### 4.3. Treatment

The treatment of choice is LT4. Treatment should be promptly initiated, within the first two weeks of life or immediately upon confirmation of serum test results. The initial dosage typically ranges from 10 to 15 µg/kg/day. For neonates with a severe form of hypothyroidism (indicated by notably low pre-treatment TT4 or FT4 values), an elevated starting dose is advisable to expedite the normalization of hormone levels and potentially enhance cognitive development. Neonates with mild to moderate hypothyroidism should begin with a lower initial dose 10 µg/kg/day. Oral administration is recommended, and the medication can be given either in the morning or evening, before or during a meal. Consistency is crucial, and the administration method should remain the same every day. The dose is adjusted based on the individual’s TSH and FT4 levels, and the correct dose is determined for each specific case [2,13].

### 4.4. Monitoring

Serum/plasma FT4 (or TT4) and TSH concentration should be regularly monitored. Sample collection should occur at least 4 h after the last LT4 administration. The recommended follow-up schedule involves an assessment at 1–2 weeks after treatment initiation. During the first year of life, intensive follow-up is advised, with evaluations every 2 weeks until TSH levels are fully normalized. Subsequently, appointments are recommended at 1–3 months until the age of 12 months. From there, reassessments should occur every 2–4 months between 1–3 years, and every 3–6 months until the completion of growth. In cases of suspected low compliance or abnormal hormone values, consider increasing the frequency of monitoring. When changing the pharmaceutical form or LT4 dose, evaluation every 4–6 weeks is recommended [2,13]. Laboratory tests provide the definitive diagnosis. For neonates, the primary challenge is to heighten the level of suspicion for metabolic or endocrine disorders, prompting early testing within the first 14 days of life.

### 4.5. Clinical Manifestation

Recently, genetic causes of HC have been described, exhibiting distinct clinical characteristics. Genetic causes of thyroid dysgenesis (TD) account for less than 5% of cases, leading to the assumption that TD is a sporadic pathology [3]. Permanent HC can be isolated or as part of a syndrome. For instance, Bamforth-Lazarus syndrome (FOXE1) is marked by thyroid dysgenesis, cleft palate, hair changes, and potentially bilateral choanal atresia or bifid epiglottis. Brain-Lung-Thyroid syndrome (NKX2-1) presents varying degrees of CH, along with respiratory issues and hereditary benign chorea. Alagille syndrome type 1 features thyroid in situ, accompanied by liver (bile duct hypoplasia) and cardiac anomalies. Other syndromes associated with CH include Williams-Beuren syndrome, DiGeorge syndrome with thyroid hypoplasia and subclinical hypothyroidism, Kabuki syndrome, and Johanson Blizzard syndrome, which includes eutopic thyroid [2,4]. In the previously presented cases, CH did not exhibit any associated phenotypic changes commonly seen in genetic diseases. This posed challenges for early diagnosis of the condition. Genetic causes of CH are characterized by specific alterations as mentioned earlier. Thyroid hormones play a crucial role in childhood development [14], with their typical clinical manifestations outlined in the table below (Table 4).

The clinical manifestations of CH originate from the inadequate response to normal thyroid hormones. However, these symptoms are not specific and can be observed in various other neonatal conditions. These include goiter formation, feeding difficulties, constipation, hypothermia, bradycardia, edema, wide fontanelles, macroglossia, prolonged jaundice, umbilical hernia, and developmental delays [4]. CH often presents in the immediate neonatal period, showing a co-occurrence with respiratory distress syndrome (RDS) due to a surfactant deficiency mechanism. This condition is further marked by persistent hypotonia, feeding difficulties, and metabolic disorders like hypoglycemia. These symptoms are frequently encountered in NICUs. Newborns with hypothyroidism are often asymptomatic at birth and are typically identified through neonatal screening. In some cases, the severe general condition and the complex treatments administered may affect the initial screening result. The suspicion of CH can arise when a newborn admitted to the NICU due to an acute initial pathology does not respond appropriately to treatment. If the newborn’s clinical condition, intestinal transit, appetite, tone, and reactivity fail to normalize despite the prescribed treatment, it could indicate the possibility of hypothyroidism. In a study conducted by Jinfu Zhou et al. [15] involving 125 newborns diagnosed with CH, several perinatal risk factors were identified. These factors include gestational diabetes, maternal thyroid pathology, and advanced maternal age as maternal risk factors. Among neonatal risk factors, female sex was associated with permanent CH, prematurity was linked to transient HC due to glandular immaturity, and postmaturity increased the risk of permanent CH. Other factors included macrosomia, congenital defects [with cardiac anomalies being the most common, showing a higher frequency in cases of CH], infections, and fetal distress (Table 5).

Both cases presented shared a common factor: maternal diabetes. Neonatal hyperthyrotropinemia has been documented in cases of gestational diabetes, in contrast to pregnancies with normal glucose tolerance. Moreover, an elevated risk of subclinical hypothyroidism during pregnancy has been observed in women diagnosed with gestational diabetes [16]. In this context, hormonal levels (TSH and FT4) in the early stages of pregnancy in women with gestational diabetes were notably lower, with reduced FT4 levels serving as an independent risk factor for gestational diabetes [18]. Thus, optimal metabolic control during pregnancy holds significant importance in mitigating maternal and fetal morbidity, including both transient and permanent neonatal hypothyroidism [16]. Regarding the cases presented, one diagnosed newborn had a maternal history of thyroid pathology. It is noteworthy that maternal conditions like these can affect not only the pregnancy duration but also have an impact on the neurodevelopment of the fetus [7]. Maternal thyroid hormones pass to the fetus through the placenta, starting in the first trimester. Fetal thyroid hormone production begins in the second trimester, with T3 binding to brain receptors by week 10, which is critical for fetal brain development. Fetal T4 levels rise progressively until term; hence, preterm infants often have reduced TSH, T3, and T4 levels [10,14]. The placenta’s role is pivotal in responding to maternal hormone levels. Studies have shown that some conditions (e.g., preeclampsia, intrauterine growth restriction, abortion) were associated with abnormal maternal thyroid hormone levels [19,20]. Thyroid hormones support trophoblast growth, differentiation; thus, they may be involved in both fetal development, placenta connection, amniotic fluid, and nutrient supply. Beyond its role in hormone transport, the placenta plays a significant role in iodine transportation. A recent study by Burns et al. suggests that the placenta acts as a storage reservoir for iodine, potentially protecting the fetus in cases of inadequate maternal iodine intake [21]. Both low maternal thyroxine concentration [7] and maternal iodine deficiency [22] have been associated with impacts on a child’s psychomotor and cognitive abilities [7,22]. Throughout pregnancy, the intricate interactions of iodothyronine deiodinases (DIO) play a crucial role in finely tuning and providing the necessary levels of thyroid hormones essential for normal fetal brain development. T4 activation is facilitated by DIO1 and DIO2, while T3 and T4 inactivation is managed by DIO3. Furthermore, DIO2 contributes to the intracellular elevation of T3 [23]. Diagnostic and intrauterine treatment methods have advanced, sometimes treating the fetus itself as the patient. Fetal thyroid issues can be managed with medication given to the pregnant woman. Few cases of fetal goiter linked to fetal hypothyroidism have been reported. The goiter can enlarge significantly, causing complications like excess amniotic fluid and difficulties in natural birth. In specialized literature, intra-amniotic thyroxine administration has been mentioned to reduce fetal goiter volume due to hypothyroidism [24].

### 4.6. Screening Protocols

- Anton-Paduraru et al. suggest testing on day 3–5 of life, or at 48 h in cases of rapid discharge. Premature infants can be tested after introducing protein into their diet [25].
- Nanu et al. recommend testing on day 3–5 for term newborns with normal weight. For premature or low birth weight newborns, or those with specific conditions, if enteral nutrition or protein solutions are absent, the test is repeated after 2 weeks or at 2 weeks of life [5].
- European Society for Pediatric Endocrinology consensus guidelines advocate screening for primary HC after 24 h, with the optimal interval being 48–72 h of life [2].

Special cases are patients with potential inconclusive or false negative initial results that require a second test at 2 weeks of life or 2 weeks after the first test. These special categories include: preterm infants, low birth weight (LBW) and very low birth weight (VLBW) infants, sick newborns admitted to NICU, samples collected within the first 24 h of life, and newborns from multiple pregnancies, particularly in the case of monozygotic twins [13,26]. This second test is necessary due to potential masking of primary HC by TSH suppression. Retesting depends on the care center’s policy; not all programs retest due to data limitations. More research is needed for this neonatal group [13]. Early screening and retesting would detect CH sooner, reducing treatment delay but increasing program costs.

## 5. Conclusions

CH is a medical condition that shows highly favorable outcomes when treated optimally. However, the critical concern within this context is the potential for neurological complications due to delayed diagnosis and treatment initiation. Elevating the level of early suspicion for this condition becomes paramount to averting complications. This objective can be accomplished through dynamic differential diagnosis in the early stages of neonatal pathology. It involves connecting the dots between the clinical evolution of the newborn, the pregnancy and fetal development, and the maternal medical history. Given the typical incidence of HC at 1 case per 3000–4000 live newborns, the occurrence of 2 cases in 2500 births raised suspicions of other potential causes. Furthermore, the recent decline in birth rates is noticeable, linked to a reduced population fertility and an increased prevalence of maternal thyroid pathology due to various factors. A pivotal aspect in the assessment and management of CH is recognizing unexpected clinical outcomes, such as delayed progress or stagnation, despite the application of suitable initial treatment. Additionally, noting instances where fetal or maternal adnexal pathology is associated without discernible underlying causes serves as a crucial clinical indicator. An important clinical aspect in testing and treating CH is recognizing unforeseen clinical outcomes. Additionally, fetal or maternal health issues without clear underlying causes can provide valuable insights.

## Figures and Tables

**Table 1:** Similarities between the cases.

	Case 1	Case 2
**Respiratory System**		
Endotracheal intubation	Yes, both at 48 h of life	
Duration of intubation (days)	13	4
Duration of mechanical ventilation (days)	5	3
Complications	Bilateral pneumothorax	No
**Digestive System**		
Emesis	Yes	Yes
Distended abdomen	Yes	Yes
Abnormal stools	Yes	Yes
**Cardiovascular System**	Small ostium secundum atrial septal defect, large aneurysm of the interatrial septum, non-hemodynamically significant persistent ductus arteriosus, moderate to severe valvular pulmonary stenosis	Moderate atrial septal defect
**Neurological System**		
Hypotonia	Yes	Yes
Abnormal sucking and feeding	Yes	Yes
**Age When Discharged (Days)**	64	32

**Table 2:** Thyroid hormones levels.

Examination	1st	2nd *	3rd *	Last in Hospital
**Patient 1**				
FT4 *	**0.96**	1.90	**3.75**	**5.46**
TSH *	**>75.0**	**39.6**	3.97	1.89
**Patient 2**				
FT4 *	**<0.30**	**0.663**	1.52	**2.33**
TSH *	**>75.0**	**>75.0**	3.44	**0.97**

* normal values: FT4 1.1–2.0 ng/dL, TSH 1.3–8.8 Uui/mL; 2nd examination—after 5 days, 3rd examination—after 8–12 days.

**Table 3:** Evolution after hospital discharge.

	Age	Body Weight	LT4 Dose *	TSH *	FT4 *
**Patient 1**	2 months and 17 days	4320 g	3.7	6.07	0.88
	3 months and 16 days	4790 g	4.5	7.3	1.16
	4 months and 8 days	5260 g	3.8	**0.51**	**2.12**
	5 months and 9 days	6270 g	2.8	**0.45**	1.44
	6 months and 11 days	6700 g	2.3	**0.6**	1.19
	7 months and 9 days	7200 g	2.2	1.6	1.15
	8 months and 12 days	7400 g	2.1	1.49	1.2
	9 months and 17 days	7700 g	2	1.47	0.99
**Patient 2**	1 month and 11 days	3600 g	5	**21**	1.63
	2 months	4500 g	5.7	**11.51**	1.51
	3 months and 5 days	6500 g	5.2	**13.4**	1.26
	4 months and 10 days	7000 g	4.5	**0.44**	1.98
	5 months and 7 days	7800 g	3.5	**0.05**	1.89
	6 months and 6 days	8100 g	3.7	6.59	1.49
	7 months and 7 days	9200 g	3.2	3.7	1.51

* dose—µg/kg, normal values: FT4 0.8–2.0 ng/dL, TSH 0.8–8.5 Uui/mL.

**Table 4:** The effect of thyroid hormones in fetal and neonatal development [7].

System/Organ	Effect
**Nervous System**	Development of astrocytes, glial cells, and synapses
**Muscles and Skin**	Development of skin and muscles
**Brown Adipose Tissue**	Activation of thermoregulation in newborns
**Liver**	Hepatic gluconeogenesis activation
**Bone**	Osseous development
**Lung**	Development of type II pneumocytes and surfactant synthesis
**Cardiovascular System**	Cardiomyocyte maturation and cardiovascular system development
**Global Development**	Birth weight and length

**Table 5:** Maternal risk factors for abnormal fetal and neonatal thyroid development.

Maternal Risk Factors	Instances
**Maternal Conditions**	Gestational diabetes [15,16,17] Thyroid pathology [4,6]
**Maternal Hypoiodemia** [4,6]	
**Maternal Hyperiodemia** [4,6]	Topical—use of iodine-based solutions [17]
	High consumption of seaweed, fish, seafood [11,12]
	Iodine nutritional supplements [4]
**Others**	Maternal age ≥ 40 years [15,16] Consanguinity [4]

## Data Availability

Not applicable.
